# Cost of exome analysis in patients with intellectual disability: a micro-costing study in a French setting

**DOI:** 10.1186/s12913-023-09373-z

**Published:** 2023-04-21

**Authors:** AL Soilly, C Robert-Viard, C Besse, AL Bruel, B Gerard, A Boland, A Piton, Y Duffourd, J Muller, C Poë, T Jouan, S El Doueiri, L Faivre, D Bacq-Daian, B Isidor, D Genevieve, S Odent, N Philip, M Doco-Fenzy, D Lacombe, ML Asensio, JF Deleuze, C Binquet, DISSEQ Investigators Group, S Arpin, P Blanchet, S Blesson, O Boute-Benejean, T Busa, E Colin, C Coubes, F Devillard, P Edery, S El Chehadeh, M Fradin, A Goldenberg, A-M Guerrot, Y Herenger, N Houcinat, N Jean-Marcais, P. S. Jouk, L Lambert, A Lavillaureix, M Legendre, B Leheup, S Manouvrier, S Mercier, S Moutton, M Nizon, L Pasquier, F Petit, L Pinson, C Poirsier, L Pons, A Putoux, C Quelin, M Renaud, M Rossi, A Sorlin, M Spodenkiewicz, J Thevenon, A Toutain, J Van-Gils, C Vanlerberghe, A Verloes, M Vincent, C Vincent-Delorme, M Willems, A Ziegler, C Thauvin-Robinet, C Lejeune

**Affiliations:** 1 CHU Dijon Bourgogne, Délégation à la Recherche Clinique et à l’Innovation, USMR, F-21000 Dijon, France; 2 CHU Dijon Bourgogne, Délégation à la Recherche Clinique et à l’Innovation, Unité Innovation, F-21000 Dijon, France; 3 CHU Dijon Bourgogne, Inserm, Université de Bourgogne, CIC 1432, Module Épidémiologie Clinique, F21000 Dijon, France; 4 Université Paris-Saclay, CEA, Centre National de Recherche en Génomique Humaine (CNRGH), Evry, France; 5 Inserm, Université Bourgogne-Franche-Comté, UMR1231, équipe GAD, Dijon, France; 6 Laboratoires de Diagnostic Génétique, Hôpitaux Universitaires de Strasbourg, Institut de Génétique Médicale d’Alsace (IGMA), 67000 Strasbourg, France; 7 Unité Fonctionnelle de Bioinformatique Médicale appliquée au diagnostic (UF7363), Hôpitaux Universitaires de Strasbourg, Strasbourg, France; 8 Inserm UMRS_1112, Institut de Génétique Médicale d’Alsace, Université de Strasbourg, France et CHRU, Strasbourg, France; 9 CHU Dijon Bourgogne, Service financier, 21000 Dijon, France; 10 CHU Dijon-Bourgogne, Centres de Référence Maladies Rares « Anomalies du Développement et syndromes malformatif de l’Est » et « Déficiences intellectuelles de causes rares », Fédération Hospitalo-Universitaire Médecine Translationnelle et Anomalies du Développement (TRANSLAD), Dijon, France; 11 Service de Génétique Médicale, CHU de Nantes, Nantes, France; 12 Département de Génétique Médicale, Centre de Référence Maladies Rares, Anomalies du Développement et Syndromes Malformatifs Sud-Languedoc Roussillon, Hôpital Arnaud de Villeneuve, Montpellier, France; 13 Service de Génétique Clinique, Centre Hospitalier Universitaire Rennes, F-35203 Rennes, France; 14 Centre National de la Recherche Scientifique Unité Mixte de Recherche 6290, Institut Génétique et Développement de Rennes, Université de Rennes 1, F-35203 Rennes, France; 15 Département de Génétique Médicale, Hôpital d’Enfants de La Timone, Marseille, France; 16 Service de Génétique, CHU de Reims, EA3801 Reims, France; 17 CRMR Anddi-Rares constitutif, CLAD-EST, CHU Reims, Reims, France; 18 CHU de Bordeaux, Génétique Médicale, INSERM U1211, Laboratoire MRGM, Université de Bordeaux, Bordeaux, France

**Keywords:** Intellectual disability, Exome sequencing, Micro-costing, Cost analysis

## Abstract

**Background:**

With the development of next generation sequencing technologies in France, exome sequencing (ES) has recently emerged as an opportunity to improve the diagnosis rate of patients presenting an intellectual disability (ID). To help French policy makers determine an adequate tariff for ES, we aimed to assess the unit cost per ES diagnostic test for ID from the preparation of the pre-analytical step until the report writing step and to identify its main cost drivers.

**Methods:**

A micro-costing bottom-up approach was conducted for the year 2018 in a French setting as part of the DISSEQ study, a cost-effectiveness study funded by the Ministry of Health and performed in collaboration with the *GAD (Génétique des Anomalies du Développement)*, a genetic team from the Dijon University Hospital, and a public sequencing platform, the *Centre National de Recherche en Génomique Humaine* (CNRGH). The analysis was conducted from the point of view of these two ES stakeholders. All of the resources (labor, equipment, disposables and reagents, reusable material) required to analyze blood samples were identified, collected and valued. Several sensitivity analyses were performed.

**Results:**

The unit nominal cost per ES diagnostic test for ID was estimated to be €2,019.39. Labor represented 50.7% of the total cost. The analytical step (from the preparation of libraries to the analysis of sequences) represented 88% of the total cost. Sensitivity analyses suggested that a simultaneous price decrease of 20% for the capture kit and 50% for the sequencing support kit led to an estimation of €1,769 per ES diagnostic test for ID.

**Conclusion:**

This is the first estimation of ES cost to be done in the French setting of ID diagnosis. The estimation is especially influenced by the price of equipment kits, but more generally by the organization of the centers involved in the different steps of the analysis and the time period in which the study was conducted. This information can now be used to define an adequate tariff and assess the efficiency of ES.

**Trial registration:**

ClinicalTrials.gov identifier NCT03287206 on September 19, 2017.

**Supplementary Information:**

The online version contains supplementary material available at 10.1186/s12913-023-09373-z.

## Background

The emergence of next-generation sequencing technologies (NGS), which can be used to sequence a large number of DNA samples in a single sequencing reaction, is a real technological breakthrough for the molecular diagnosis of rare diseases.

Among the patients who can benefit from NGS technologies, etiological genetical diagnosis of intellectual disability (ID) presents a considerable challenge. ID is the most common cause of referral in pediatric genetic centers. It is a neurodevelopmental disorder characterized by impaired intellectual performances and dysfunctional adaptive behavior, affecting between 1 to 3% of the general population; approximately 15 out of 1000 people have mild ID and 3 out of 1000 have severe ID [1]. In France before the arrival of NGS in the clinic, diagnosis was based on clinical expertise, the use of Chromosomal Micro-array Analysis (CMA) to detect chromosomal copy number variations (CNV), the search for X-fragile syndrome (FRAXA analysis), and, if necessary, the study of targeted genes based on clinical data [2]. With the development of NGS, the genes already known to be involved in genetic conditions can now be simultaneously analyzed [3]. NGS is also used for exome sequencing (ES), which can determine the variations in all coding regions (exons) in a single scan of known genes [4, 5]. Data from the literature showed a higher diagnostic yield for ES (42%) compared to gene panels (32%), providing arguments in favor of routine ES [6, 7].

But beyond the evidence of the higher diagnostic performance of ES, estimating its production cost is also essential to inform French health authorities about ES affordability before its diffusion in clinical practice as ES is becoming a routine examination in Europe [8, 9]. These data could also be used to help determine a relevant tariff to be covered by the French collectivity, which does not exist at the moment and help assessing the sustainability of the reimbursement of such a diagnostic test for identifying the ID etiology.

In this context, the French Ministry of Health funded a multicenter prospective cost-effectiveness analysis (DISSEQ study) as part of an economic research program. The aim was to evaluate the efficiency of ES for the diagnosis of ID in a cohort of 330 patients without prior genetic investigations. An essential element of this study is the estimation of the unit production cost per ES diagnostic test for ID. This estimation is all the more important that, to the best of our knowledge, there is no French cost data in the field of ID. The only result published in France concerned the field of oncology [10]. It was estimated to be €1,608 per patient in 2018 (considering two samples per patient: one from the tumor and one from nontumoral tissue). In addition, this result needs to be confirmed as the data was collected in only one care center and the unit cost per ES was calculated by combining a micro-costing and gross-costing method. At an international level, a literature review published in 2018 showed that the cost of ES for neurological or neurodevelopmental disorders diagnosis ranged from €792 to €3,000 for a single test (the index case only) and was estimated to be €3,600 for a trio (the index case and the two biological parents) [11–18]. The large range of estimations may be explained by differences in pricing but also in the organization of the biology, sequencing and bioinformatics centers and in the pre-analytical protocols used. Both the fact that it is difficult to extrapolate cost estimations into other contexts and the need for results based on French data in the field of ID justified our goal to estimate the unit cost per ES diagnostic test, from the preparation of the pre-analytical until the report writing in patients presenting ID and to identify its main drivers.

## Methods

### Summary of the DISSEQ study

Briefly, the DISSEQ study (ID RCB: 2016-A00350-51- NCT03287206) is a cost-effectiveness analysis in which each included patient is his own control. The 330 included patients coming for a first genetic consultation benefited from the three main strategies compared, in parallel: 1/ CMA, FRAXA analysis and panel sequencing of 459 genes (459GPS), 2/ CMA, FRAXA analysis ± the analysis of clinically oriented targeted genes ± paraclinical exams according to clinical orientations, 3/ ES and FRAXA analysis. After providing information and obtaining consent, blood samples were collected from the patient in each of the nineteen participating care centers. CMA, FRAXA analysis and clinically oriented targeted genes analyses were performed in laboratories most often located at the same site as the inclusion center. Concerning 459GPS and ES, samples of the index case were mailed to the “Genetics of Developmental Abnormalities” (GAD) laboratory team (Inserm U1231) of the Dijon University Hospital. The extracted DNA from the only index case was then sent for 459GPS and ES sequencing analysis at the “Centre National de Recherche en Génomique Humaine” (CNRGH), a national research center in Évry-Courcouronnes that has been part of the CEA (Commissariat à l'Énergie Atomique et aux Énergies Alternatives) since 2008. The CEA addresses scientific questions requiring high throughput sequencing through the development and implementation of innovative and integrated technologies. The platform carried out the capture and sequencing of the 459GPS and the ES, and then transferred for the bioinformatics analyses the raw data from the 459GPS to the Strasbourg molecular genetics laboratory team and from ES to the Dijon GAD team. The validation of the candidate variations, as well as the interpretation of the results by biologists, was then carried out in each of the two teams. At Dijon, a multidisciplinary meeting was organized for ES. Once the results of 459GPS and ES were available (within 9 months), a joint "research" report combining the results drawn up by the Strasbourg and Dijon teams was sent to the geneticist who included the patient in the study. Three types of results could be obtained at the end of the study for each strategy (positive result defined by the identification of a 4 or 5 class variant according to the American College of Medical Genetics: the diagnosis of ID was made; negative result: the diagnosis of ID was not made; non-conclusive result: a genetic abnormality has been found, but a clear diagnosis could not be made). Results were then delivered to the patient and his or her parents. Within this framework, it was planned to conduct the cost-effectiveness analysis (on going data collection and checking) from the health care system point of view (care providers) within the time horizon of 9 months. The efficiency criterion will be based on the estimation of an incremental cost-effectiveness ratio, expressed in terms of cost per additional positive causal diagnosis. Only direct medical costs including the cost of all genetic analyses but also complementary and confirmatory medical examinations will be considered.

### Micro-costing study design

Within the framework of this planned cost-effectiveness study, a bottom-up micro-costing approach was used to estimate the unit cost per ES diagnostic test for ID for the year 2018. This method consists in identifying all resources (inputs) consumed to analyze blood samples and to value them using their unit price [19, 20]. The choice of this method is justified by the innovative character of ES [21] and the need to provide information to French policy makers to determine a permanent tariff for ES. The micro-costing analysis was conducted from the point of view of the providers, i.e. from the two centers involved in the ES analysis process (the GAD team and the CNRGH).

### ES analysis process

**Fig. 1:**
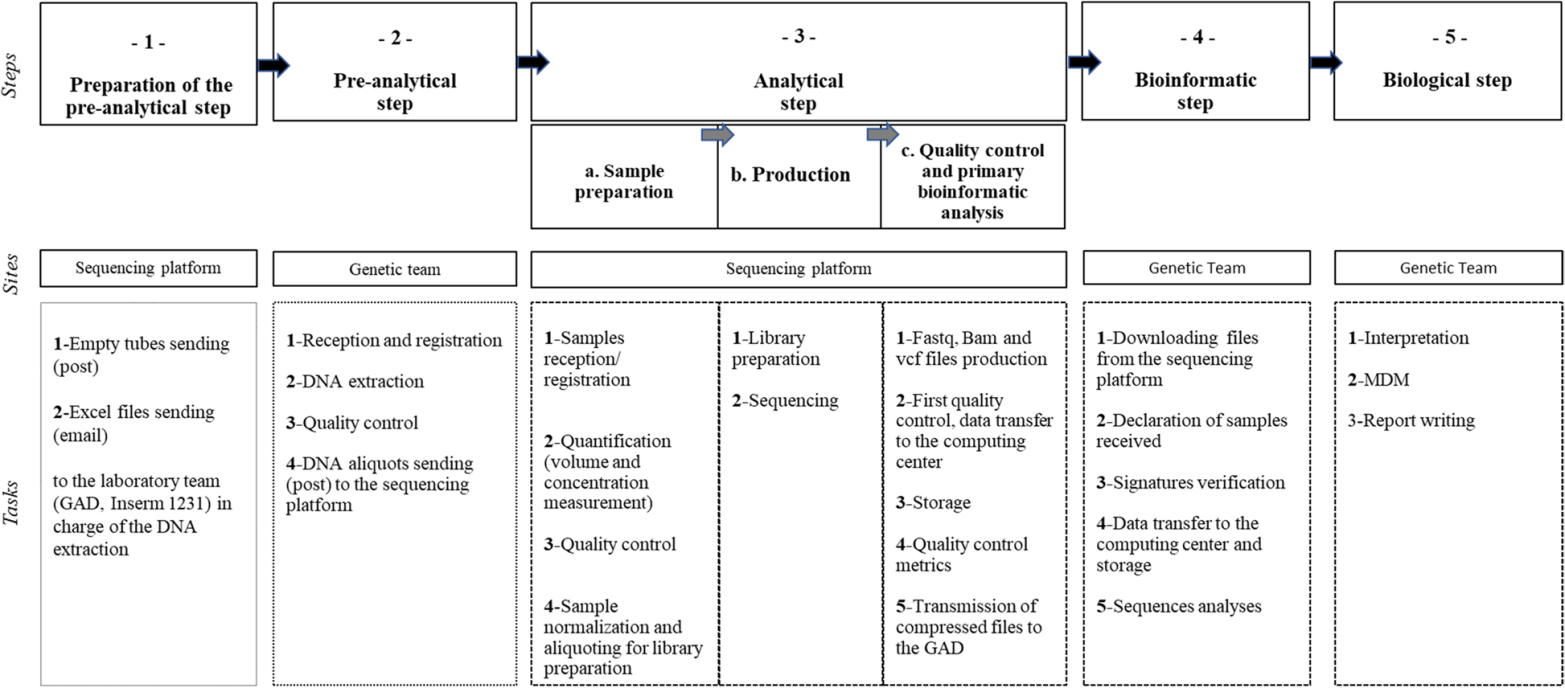
The ES process. Represents the ES process with the five steps of the analysis: the preparation of the pre-analysis, the pre-analytical, the analytical, the bioinformatics steps and the biological step

The time horizon for the cost estimation started from the preparation of the pre-analytical step (step 1) until the report writing (step 5), about 9 months (Fig. 1). The five steps are as follows:

- *Step 1 (preparation of the pre-analytical step)*: corresponding to the sending of empty tubes (by post) and formatted files (by email) from the CNRGH sequencing platform to the GAD, which was in charge of the pre-analytical step.
- *Step 2 (pre-analytical)*: conducted at the GAD, and including DNA extraction, quality control, and shipment of the DNA aliquots to the CNRGH sequencing platform.
- *Step 3 (analytical)*: carried out by the CNRGH sequencing platform and divided in three steps: 1/ sample preparation (including sample reception and registration, storage, quantification, quality control and normalization before production); 2/ production (including library preparation and sequencing); 3/ quality control and primary bioinformatics analysis (including the production of fastq, bam and vcf files, first quality control and data transfer to the computing center and storage, determination of sequence quality control metrics, and transfer of compressed files to the GAD for the bioinformatics step).
- *Step 4 (bioinformatics)*: performed by the bioinformatician of the GAD, including the download of files from the platform, the declaration of samples received, the signature verification, the transfer of data to the computing center and the storage facility, and finally the sequencing and bioinformatics analysis which consists in prioritizing and annotating gene variations already associated with ID and/or other developmental abnormalities according to Online Mendelian Inheritance in Man (OMIM) database references.
- *Step 5 (biological step):* including the interpretation and, if required, a bibliographic research by a certified biologist, followed by a multidisciplinary meeting (MDM)- gathering health practitioners and aiming at discussing the clinical situations—which lead to the drafting of a report indicating the results.

## Cost estimations

### Identification of resources

The categories of resources included consumables (disposable materials and reagents), reusable laboratory materials, equipment (machines and computers), and labor. Maintenance of equipment and overheads were also considered (Supplementary Information).

The entire pre-analytical process (step 2) was prospectively described by direct observation of the GAD team by a working group composed of two health economists and a clinical research technician. This process and the associated resources to be collected were then recorded on a grid that was validated by a biologist from the GAD. All the data for 10 consecutive patients issued from the 330 patients of the DISSEQ study were then collected by two biological technicians separately (5 samples each) during a typical working day in order to consider inter-operator variability in lab handling.

The description of steps 1, 3 and 4 were based on detailed descriptions provided by the biologists and bioinformaticians involved in these processes. A specific grid was then designed, validated and completed during a typical working day. For the step 5, the time required to interpret data (including a bibliographic research when applicable), the duration of the MDMs (including their preparation, the presentation of the clinical cases and the discussion time), the number of health professionals attending the MDMs, as well as the time required to write the report were collected and entered into the electronic case-report form of the DISSEQ study by the biologist of the GAD team.

### Resource valuation

*Disposable materials and reagents* were used in steps 1 to 3. Quantities of reagents (such as reagent kits and lysing solutions, expressed in ml or μl) and materials (such as barcode labels, envelopes, tags, gloves, tips, tubes, strips, optical films, and quantification plates, expressed in numbers) were valued by their unit price. The unit price was defined as the total purchase price (including all taxes) paid by the GAD and the CNRGH respectively to their providers, divided by the total purchased quantities.

*The reusable laboratory materials* (e.g. pipettes) used in steps 2 and 3 were not specifically dedicated to the DISSEQ study. Therefore, we calculated a dedicated allocation rate to be applied to each material. This rate was defined as the number of tubes used for DISSEQ (330) divided by the total number of tubes processed during each step in 2018 -year in which the 330 patients were included in the study- (2,813 at the GAD, 37,500 at the CNRGH sequencing platform for the sample preparation step, 8,244 for the library preparation step and 4,096 for the sequencing step). This rate was then applied to the annual depreciated purchase price of each of the materials used. It was also applied to maintenance fees.

*Equipment* included machines (used in steps 2 and 3), and computers and software (steps 2 to 4). A similar allocation rate was applied to the depreciated annual price of each of the machines (*e.g*. in step 2: centrifugal machine, Vortex, Multiscan, GelDoc; in step 3: sequencing machine Hiseq4000, pipetting robots, Tapestation4200 Agilent, thermocycling machine…). Concerning computers, the rate was based on the time mobilized for declaring samples, launching transfer or calculation orders, divided by the legally defined hours worked per year for a hospital engineer (1,720 h in French law). Software was either included in the cost of the equipment or was free access. Equipment also included the costs of data storage and the computer calculations. Storage costs at steps 3 and 4 were based on the fees paid by the CNRGH sequencing platform to the CEA (corresponding to 60 Giga octets per sample) and by the GAD to the Burgundy University Calculation Center (CCuB) (corresponding to 20 Giga octets per sample), respectively. Computer calculation costs were also based on the annual fees paid respectively by the CNRGH sequencing platform to access 120,000 computing hours (corresponding to 300 Central Processing Unit [CPU] hours per sample) and by the GAD for one million computing hours (corresponding to 50 h per sample).

There are no specific guidelines concerning the use of a depreciation rate. We followed the rules used by the hospital of Dijon, corresponding also to the French government guidelines (https://bofip.impots.gouv.fr/bofip/4520-PGP.html/identifiant%3DBOI-BIC-AMT-10-40-30-20130923). Thus, they were applied linearly and were as follow: 50% for reusable materials, 15% for machines and 20% for computers.

*Labor:* staff costs were calculated according to the time spent (in minutes) on each task by each category of staff (secretary, technician, engineer, bioinformatician, biologist, clinician, technical manager), and valued by the average charged gross salary associated with their respective work place: at the Dijon hospital for the GAD; at the CEA for the sequencing platform of the CNRGH. Concerning step 5 (biological step), the time required to interpret data by a certified biologist for the DISSEQ samples was averaged and valued by the average charged gross salary of a biologist. Concerning MDMs which were organized at Dijon, their average duration was calculated. We also considered the number of health professionals participating to these MDMs. Their number could vary from four to ten. Because their working status (hospital practitioner, specialized assistant, assistant professor, associate professor, professor, etc.) was not known, the median number of participants was calculated and valued by the average charged gross salary of a hospital practitioner. Similarly, the average charged gross salary of a hospital practitioner was used to value the time associated to the report writing.

*Maintenance costs* were only relevant for some types of equipment. For steps 2 and 3, the cost was valued according fees paid by the GAD and the CNRGH sequencing platform to their external providers. For step 4, maintenance concerned the pipeline and corresponded to the time spent by the bioinformatician, valued by the average gross salary of his/her position at the Dijon hospital.

*Overheads* corresponded to the costs that could not be directly attributed to one specific task. These generally include the cost of electricity and water consumption, insurance, and depreciation of buildings. Overheads were applied to all cost items (disposables and reagents, equipment and labor), except maintenance in steps 2 and 3, and the storage and calculation costs in step 4 because they were already included in the fees paid to the external providers. For step 4 particularly, overheads only concerned the computers and the time mobilized by the bioinformatician for the maintenance of the pipeline. A rate of 20% was used [14, 22].

The total cost per diagnosis test was calculated for the whole ES analysis process by adding up all of the estimated costs from step 1 to 5. No discounting was applied given the time horizon of the micro-costing study.

#### Assumptions

To calculate all unit costs, we made the following assumptions: 1/ there is a continuous and unlimited access to disposable material, reagents and small reusable laboratory equipment, 2/ the purchase prices of all the resources consumed are invariant, whatever the volume of activity, 3/ reusable materials and equipment are depreciated on a linear basis, 4/ there are no breakdowns or deterioration requiring additional investment in material and equipment, 5/ equipment is not interchangeable (i.e. the fact that several machines could alternatively do the same task was not considered), 6/ one operator is dedicated to one task (no task sharing), 7/ all employees are employed full-time (35 h per week), 8/ the activities of GAD Inserm U1231 and the CNRGH sequencing platform are not dedicated to the DISSEQ study alone, 9/ there is no re-analysis of ES data.

### Sensitivity analysis

In order to test the robustness of the results, several deterministic sensitivity analyses were performed. Most of the analyses were based on the expertise of the biologists and managers working for the GAD and the CNRGH sequencing platform: first, the cost of reagent kits in the preanalytical step were simultaneously decreased by 20% (QiAmp kit for DNA extraction, and Qubit kit for quality control). A similar analysis was performed for the analytical step with a simultaneous decrease of 20% for the capture kit (library preparation step) and 50% for the sequencing support kit. The activity volume of the DISSEQ study was also increased by 30%. We also considered the variations of interpretation time, MDM preparation and discussion as well report writing in a simultaneous way, making them range from their minimum to their maximum.

In addition, to account for inflation from 2018 to 2022 in France, we simulated a price increase based on the Consumer Price Index (CPI) calculated from January 2018 until October 2022 and corresponding to an 11.5% increase. We also considered salary enhancement measures taken in France from 2020 which was applied to all public hospital staff (https://www.gouvernement.fr/upload/media/default/0001/01/2020_07_dossier_de_presse_-_signature_des_accords_du_segur_de_la_sante_-_13.07.2020.pdf and https://www.legifrance.gouv.fr/jorf/id/JORFTEXT000046026212) (Additional file 1).

We did not consider increase in prices because of the decline in the cost of sequencing in the last 15 years [23].

## Results

### Cost estimates

**Fig. 2:**
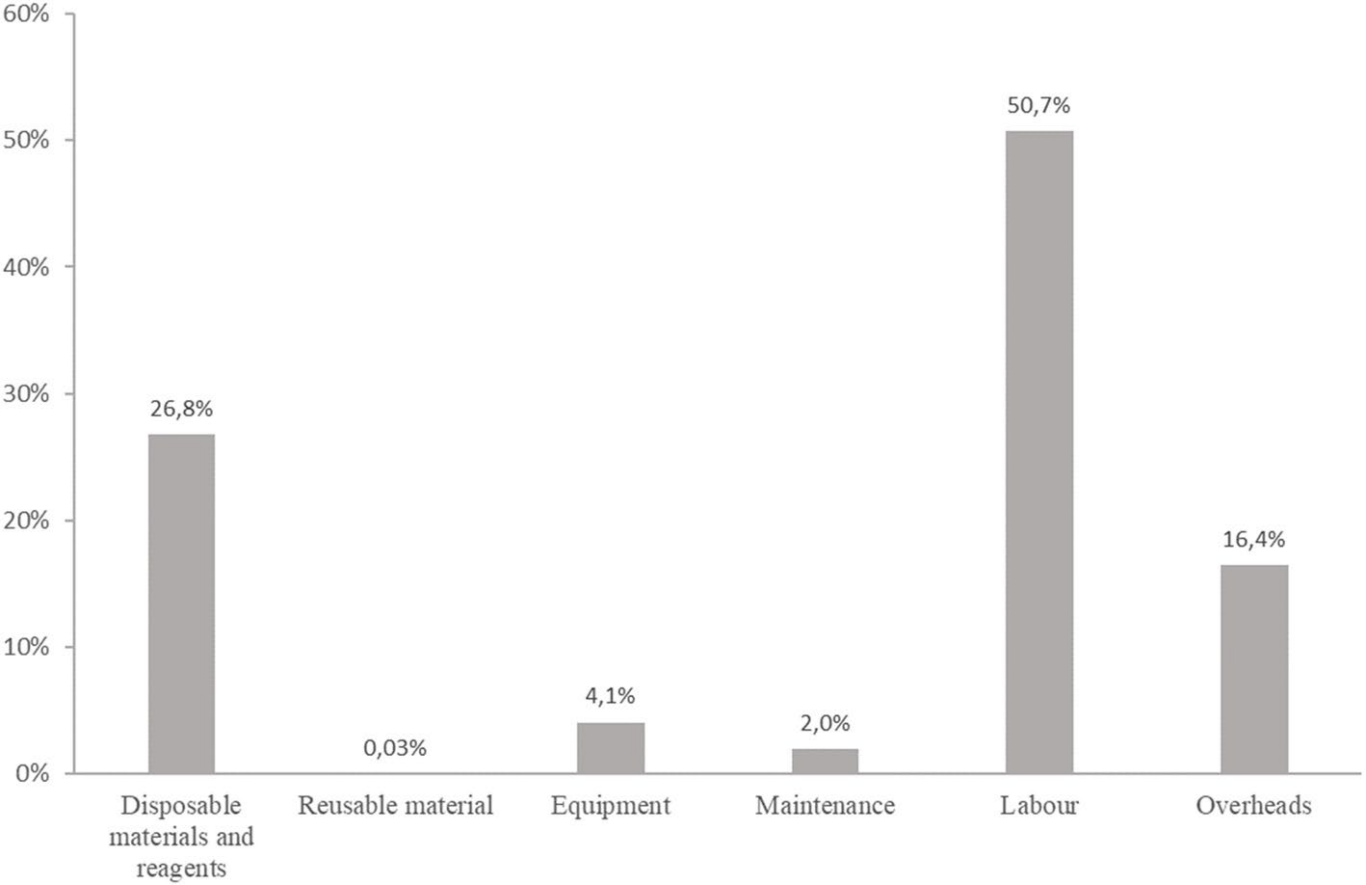
Components of the cost per ES diagnostic test for ID (%). Represents the distribution of the components (disposable materials and reagents, reusable materials, equipment, maintenance, labor and overheads) within the ES cost analysis

The unit nominal cost per ES diagnostic test for ID was estimated to be €2,019.39 (Table 1). Labor was 50.7% of the total cost, followed by the disposable materials and reagents (26.8%), and equipment (4.1%). The analytical step generated the greatest part of the total cost per diagnosis (88%), followed by the pre-analytical steps (4%). Results are presented in Fig. 2.

**Table 1:** Unit cost (€) per ES diagnostic test for ID

Resources	**- 1 -** **Preparation of the pre-analytical step**	**- 2 -** **Pre-analytical** **step**	**- 3 -** **Analytical step**			**- 4 -** **Bioinformatics step**	**- 5 -** **Biological step**	**Total**
			a.Sample preparation	b. Production	c. Quality control and primary bioinformatics analysis			
Disposable materials and reagents	€ 0.80	€ 15.59	€ 8.52	€ 515.67	-	-	-	€ 540.58
Reusable materials	-	€ 0.35	€ 0.01	€ 0.22	-	-	-	€ 0.59
Equipment	-	€ 3.42	€ 0.91	€ 44.52	€ 30.17	€ 3.15	-	€ 82.18
Maintenance	-	€ 0.72	€ 0.65	€ 21.79	-	€ 16.69	-	€ 39.86
Labor	€ 29.21	€ 47.30^b^	€ 166.13	€ 643.55	€ 57.74	€ 10.35	€ 69.71^b^	€ 1,023.99
Overheads^a^	€ 6.00	€ 13.33	€ 35.12	€ 240.79	€ 17.58	€ 5.41	€ 13.94	€ 332.18
**Total**	**€ 36.00**	**€ 80.72**	**€ 211.35**	**€ 1,466.56**	**€ 105.50**	**€ 35.60**	**€ 83.65**	**€ 2,019.39**

^a^Overheads (20% rate) applied on all resources of steps 1,2,3 and 5 excepted the maintenance. At step 4, overheads were applied on a part of equipment cost (€0.03 over the €3,15, corresponding to computer cost) and on the totality of the maintenance because it is realized by an bioinformatics engineer

^b^Labor cost for the pre-analytical step was estimated from the time spent by two biological technicians separately during a typical working day to analyze 5 index cases each, allowing the calculation of an average cost. Concerning the biological step, the cost of labor was estimated from the mean time mobilized for interpretation of data, the preparation of the meeting, the presentation and the discussion

### Sensitivity analyses

**Fig. 3:**
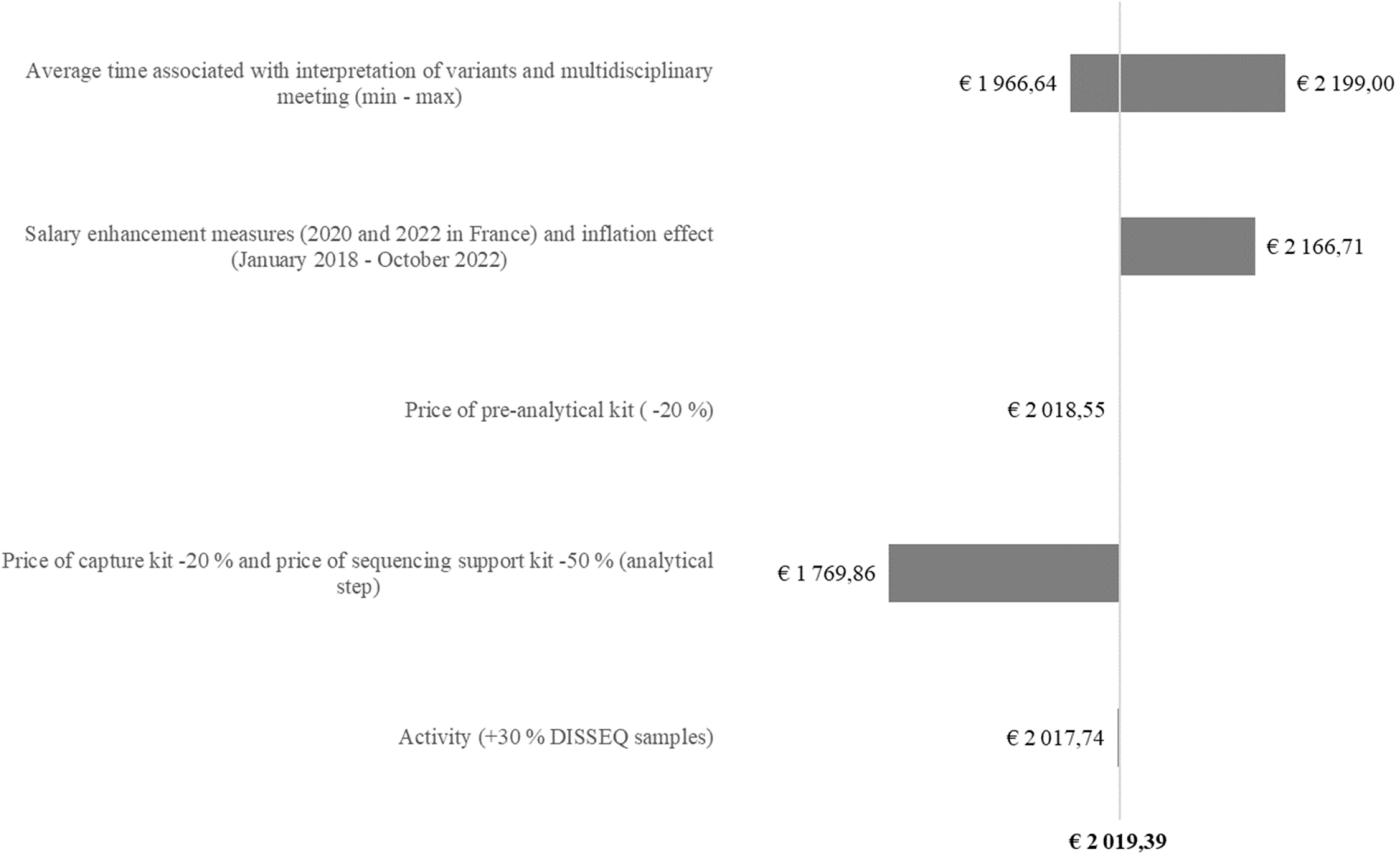
One-way sensitivity analyses. Represents the results of several one-way sensitivity analyses and shows how the baseline estimation of the cost per ES diagnostic test for ID (€ 2,019.39) varies according to key parameters

The sensitivity analyses showed that the unit cost per ES diagnostic test for ID was not changed considerably by a 20% decrease in the price of kits for the preanalytical process or the variation in activity volume (€2,018.55) (Fig. 3). A decrease in the kits’ prices for the sequencing step had a greater impact since the cost decreased by 12.3% to reach €1,769.86. The unit cost per ES diagnostic test was also influenced by the time associated with the interpretation of variants and MDM. The variation of these parameters between their minimum and maximum leads to a unit cost ranging between €1,966.64 and €2,199. Finally, the impact of CPI and salary enhancement measures impacted the unit cost which increased by 7.3% to reach € 2,166.71.

## Discussion

This study is the first to present the comprehensive unit cost per ES diagnostic test for ID in France. At the moment, there is no tariff for ES. The tariff of CMA is fixed at € 550 (code B034 in the French insurance system database) and at €135 for FRAXA (code B4050). As a comparison, 459GPS is financed via a temporary tariff of €2,209 (code N352 of the RIHN- le Référentiel des actes innovants hors nomenclature de biologie et d’anatomopathologie-) corresponding to the analysis of the biggest genes panel (> 100 kb and < 500 kb). The RIHN, set up by the French Ministry of health in 2015, is an early and transitional funding system of innovative procedures in the framework of a specific budget dedicated to research, training and innovation (https://solidarites-sante.gouv.fr/systeme-de-sante-et-medico-social/innovation-et-recherche/rihn). This €2,209 tariff includes 1/the pre-analytical control of the patient’s file, 2/the clinico-biological discussion to check the relevance of the prescription, 3/the registration of the patient's clinical data in the computer databases, 4/the nucleic acid extraction, assay and quality control, 5/the preparation, amplification and sequencing of the library, 5/the interpretation of NGS data (bioinformatics analysis, database interrogation and use of prediction software), 6/the confirmation of the identified mutation(s) regardless of the method and the study of familial segregation where possible, 7/the analysis of bibliographic databases and comparison with clinic biological data, and 8/the drafting of an explicit diagnostic report [24]. For the year 2018, we estimated the unit cost per ES diagnostic test for ID at €2,019.39, a result close to the existing 459GPS tariff.

The main strength of our study lies in the very detailed description of all the steps of the analysis process and the consideration of the resources consumed during the pre-analytical, analytical, bioinformatic phase and until the report writing, either by direct observation or based on staff interviews. Microcosting is the most accurate method for estimating the production cost of the implementation of a healthcare technology [19, 21, 25]. We used a bottom-up approach, which is the most frequently used method. It is also the methodological gold standard in the literature since it uses the monetary valuation of inputs collected at an individual level [26]. Most published cost estimations for ES analyses have used a similar approach [13–16]. Others modelled the cost based on the literature data [12], or used commercial prices [27, 28]. Some studies did not provide details about the methodology, making comparisons between results quite difficult [29, 30]; moreover they do not concern the field of neurology or neurodevelopment and involve specific issues in terms of interpretation [31].

Half of our unit cost per ES diagnostic test was composed of labor cost (50.7%) and almost one third was composed of disposable materials and reagents (26.8%). Three quarters of the cost of ES was generated in the analytical step. Our estimation is within the wide range of previously published results, which vary between €792 [15] and €3,000 [13] for a single test. Despite the fact that all settings in France and other countries follow the five main steps (from pre-analytical to report writing), caution is required when making comparisons between these results [32]. Cost estimations are highly dependent on the equipment of the laboratories and platforms and the time period considered: their size (small or large), the type of equipment and their activity volume during the time period considered (2018 in our case) can strongly influence the price of supplies and generate economies of scale. Similarly, the status of the center (academic health care provider or commercial) results in differing regulatory and quality assurance process. In our study, the CNRGH sequencing platform that prepared the libraries, sequenced DNA and conducted the primary bioinformatics analysis by providing the fastq, bam and vcf files was a research facility with regular and systematic quality control procedures implemented during the process in order to improve the quality of the data, leading to the intervention of several expert staff members at each step of the process. This specific organization, whose aim is to maximize the quality of the results, could explain why labor represented 50.7% of the total cost of ES in our study. In Tslipova, et al. [33], the estimated cost per autism spectrum disorder sample was $1,655 CAD (€ 1,220); labor made up only 19% of the total cost. This low proportion compared to our results could be partly explained by the fact that periodic validation and quality control were not included in their analysis [33]. Different practices, for example various degrees of automation in the pre-analytical step, could also explain the variable proportion of labor in the cost estimation. Similarly, the use of different generations of sequencing machines could explain certain differences. In our study, sequencing was done with the Illumina® (San Diego USA) Hiseq4000 with at least 90 to 100X coverage. Tslipova, et al. [33] and Monroe, et al. [16] used a HIseq®2500 with an average 93X coverage level, and Van Nimwegen, et al. (2016) used a HIseq®2500 with 70X coverage [15].

In a French study published by Bayle, et al.in 2021 in the field of oncology, the ES cost was estimated to be € 1,608 per patient in 2018 (considering two samples per patient). If consumables were the main driver (62% of the cost per sample *vs* 26.8% in our study), the absolute amount per sample was close (€501 vs €540 in our study). The second driver was overheads (20% *vs* 16.4%), followed by labor (15% *vs* 50.7%) and equipment and maintenance (3% *vs* 6.1%) [10]. Lower equipment and maintenance costs (€25 *vs* €122 in our study) could be explained by the fact that they did not fully include storage costs. One explanation of their lower labor costs (€119 *vs* €1,023 in our study) can also lie in their use of Illumina NovaSeq 6000sequencer. One final explanation for the differences between their results and ours could be the differences in the sequencing process of a tumor tissue compared to blood.

Methodological choices such as the choice of the costing method (top-down *versus* bottom-up gross-costing or micro-costing, use of commercial prices) may also influence the results [19]. Bayle, et al. used the gross-costing method which consisted in allocating the annual labor budget to the dedicated time of staff to each ES run instead of timing precisely the time dedicated to each step as we did in our study. This could also explain one part of the labor cost discrepancy. Because micro-costing analyses have no standardized requirements, practices can vary greatly from one study to another according to the data collection method, the choice of unit price per input mobilized, as well as the choice of cost components and how they are presented in the article [25], therefore limiting the transferability of the costs’ results from one setting to another. For instance, the steps considered to be part of the ES process were quite different from one study to another, with certain studies including patient registration [16], blood sampling [14], genomic consultation [12], sample preparation [15] interpretation [13], reporting of results [13–15] and/or the return of the results to the family [12, 16]. In the present study, costs were estimated starting at sample preparation (DNA extraction and quality control) and finishing with report writing after the MDM. We only excluded the time spent by clinicians collecting clinical data during consultations and the results’ disclosure to the parents as an associated tariff exists.

The choice of components to include in the estimate can also make comparisons challenging. For instance, we chose not to include the pipeline research and development undertaken by the GAD and the CNRGH sequencing platform. Ad hoc teams and resources were required for these activities, so their inclusion would have resulted in an overestimation of routine ES analysis. However, we included pipeline maintenance because it is a yearly occurrence. Secondly, the pipelines were not developed specifically for the DISSEQ study. This point raises questions about how to best allocate global costs to one sample (or a trio samples) when unit prices cannot be determined directly. We used two allocation keys, the first based on the number of DISSEQ samples divided by the total activity of each center for 2018, which was the year that the 330 patients were included in the study. This allocation key concerned reusable materials and machines. This choice was justified by our need to calculate a cost per sample (or per trio). When tasks required by a type of equipment were similar whatever the number of samples, which was the case of computers, another key was applied, based on the time required for declaring samples, launching transfers, or calculations, divided by the legally defined 1,720 annual working hours of a hospital engineer. Alternatives have been used in the literature, such as the number of use for all patients in the institution according the following formula (1 or 2 uses/ all the tests per year) [33] or a per-minute rate [10, 13], which reveals the lack of consensus in the methods and the complexity of micro-costing. One main limit in this study was the fact that the dedicated rate is influenced by the volume of tubes processed during the study period. 2018 was not representative of standard activity at the GAD since 1,745 tubes were processed in 2016; 3,175 in 2017; 2,813 in 2018 and 3,990 in 2019. These variations explain the reason why a sensitivity analysis was conducted. Results showed the cost of ES was not impacted (€ 2,017.74) after a 30% increase, but this result was probably largely underestimated because the increase in activity was not associated with the expected decrease in the price of some materials and supplies.

The stability of prices regardless of activity volume was one of the strong hypotheses of our study. The sensitivity analyses we performed on the price of some of the kits during the preanalytical step did not reveal a great effect contrary to the kits for the CNRGH sequencing platform. When the price of capture kit was decreased by 20% and the sequencing kit by 50% during the analytical step, the cost of ES per diagnosis was reduced by 12.3% (€1,769.86 instead of €2,019.39). We also assumed that there was an absence of exchangeability between equipment, but we estimate that the total cost would not have been strongly impacted. Similarly, excluding already depreciated equipment (refrigerators, microwave ovens, etc.) from the analysis had a small impact on the total cost. Conversely, our results are subject to change quickly with the use in the future of new equipment and consumable whose cost will be quite lower and consequently will increase the proportion linked to labor costs in the final cost.

Though there are methodological differences among the various studies that have been conducted, there are also similarities in the results. Overall, the sequencing step represented the largest proportion of the total cost (74 and 78% in Sabatini, et al. and Monroe, et al., respectively, 88.3% in our analysis) [13, 16]. In Tslipova, et al., supplies represented 39.7% of the total cost, compared with 23% in Sabatini et al. and 33% in ours (disposable materials and reagents) [13, 14].

To conclude, this study is the first to provide a comprehensive evaluation of the unit cost per ES diagnostic test for ID for ID, in a given period (2018). This estimation was conducted in the specific context of a cost-effectiveness evaluation funded by the Ministry of Health and performed in collaboration with the GAD and the CNRGH. Comparisons with previous results show that these estimations are highly influenced by the organization of the centers. Methodological choices (costing methods, extensiveness of the analysis process, allocation keys, components of the cost) also influence the results. All these differences should encourage authors to be as transparent as possible about their methodological choices, the description of the setting where the evaluation is conducted and the results reporting which should be presented as precisely as possible (by expense items and steps). Our estimation can help inform the decision maker about the tariff to be fixed for ES analysis and to estimate the cost-effectiveness ratio of ES. It is also an important reference for the calculation of other costs, particularly the whole cost of genome sequencing, for which a study is ongoing in France as part of the French Genomic Medicine Plan 2025 (PFMG) [34].

## Supplementary Information

**Additional file 1.** Resources used (year 2018). Full details of resources used. 

## Data Availability

Data generated and analyzed during this study are included in the supplementary information file.

## Abbreviations

CCuB: Burgundy University Calculation Center
CEA: Commissariat à l’Énergie Atomique et aux Énergies Alternatives
CMA: Chromosomal Microarray Analysis
CNRGH: Centre National de Recherche en Génomique Humaine
CPU: Central Processing Unit
ES: Exome sequencing
GAD: Génétique des Anomalies du Développement
ID: Intellectual disability
MDM: Multidisciplinary meeting
NGS: Next-generation sequencing
OMIM: Online Mendelian Inheritance in Man
PFMG: French Genomic Medicine Plan
