# Supplementary material for: Cost of exome analysis in patients with intellectual disability: a micro-costing study in a French setting
Source: BMC Health Serv Res. 2023 Apr 21;23:386. doi: 10.1186/s12913-023-09373-z (PMC10120135; doi:10.1186/s12913-023-09373-z)
Supplement: Supplementary file 1 — Additional file 1. Resources used (year 2018). Full details of resources used. [file 12913_2023_9373_MOESM1_ESM.docx]

**Cost of exome analysis in patients with intellectual disability: a micro-costing study in a French setting**

AL Soilly^1,2^, C Robert‑Viard^2,3^, C Besse^4^, AL Bruel^5^, B Gerard^6^, A Boland^4^, A Piton^6^, Y Duffourd^5^, J Muller^6,7,8^, C Poë^5^, T Jouan^5^, S El Doueiri^9^, L Faivre^5,10^, D Bacq‑Daian^4^, B Isidor^11^, D Genevieve^12^, S Odent^13,14^, N Philip^15^, M Doco‑Fenzy^16,17^, D Lacombe^18^, ML Asensio^3^, JF Deleuze^4^, C Binquet^3^, DISSEQ Investigators Group, C Thauvin‑Robinet^5,10^ and C Lejeune^3*^

^1^ CHU Dijon Bourgogne, Délégation à la Recherche Clinique et à l’Innovation, USMR, F-21000, Dijon, France

^2^ CHU Dijon Bourgogne, Délégation à la Recherche Clinique et à l’Innovation, Unité Innovation, F-21000, Dijon, France

^3^ CHU Dijon Bourgogne, Inserm, Université de Bourgogne, CIC 1432, Module Épidémiologie Clinique, F21000, Dijon, France

^4^ Université Paris-Saclay, CEA, Centre National de Recherche en Génomique Humaine (CNRGH), Evry, France

^5^ Inserm, Université Bourgogne-Franche-Comté, UMR1231, équipe GAD, Dijon, France

^6^ Laboratoires de Diagnostic Génétique, Hôpitaux Universitaires de Strasbourg, Institut de Génétique Médicale d’Alsace (IGMA), 67000, Strasbourg, France

^7^ Unité Fonctionnelle de Bioinformatique Médicale appliquée au diagnostic (UF7363), Hôpitaux Universitaires de Strasbourg, Strasbourg, France

^8^ Inserm UMRS_1112, Institut de Génétique Médicale d’Alsace, Université de Strasbourg, France et CHRU, Strasbourg, France

^9^ CHU Dijon Bourgogne, Service financier, 21000, Dijon, France

^10^ CHU Dijon-Bourgogne, Centres de Référence Maladies Rares « Anomalies du Développement et syndromes malformatif de l’Est » et « Déficiences intellectuelles de causes rares », Fédération Hospitalo-Universitaire Médecine Translationnelle et Anomalies du Développement (TRANSLAD), Dijon, France

^11^ Service de Génétique Médicale, CHU de Nantes, Nantes, France

^12^ Département de Génétique Médicale, Centre de Référence Maladies Rares, Anomalies du Développement et Syndromes Malformatifs Sud-Languedoc Roussillon, Hôpital Arnaud de Villeneuve, Montpellier, France

^13^ Service de Génétique Clinique, Centre Hospitalier Universitaire Rennes, F-35203, Rennes, France

^14^ Centre National de la Recherche Scientifique Unité Mixte de Recherche 6290, Institut Génétique et Développement de Rennes, Université de Rennes 1, F-35203, Rennes, France

^15^ Département de Génétique Médicale, Hôpital d’Enfants de La Timone, Marseille, France

^16^ Service de Génétique, CHU de Reims, EA3801, Reims, France

^17^ CRMR Anddi-Rares constitutif, CLAD-EST, CHU Reims, Reims, France

^18^ CHU de Bordeaux, Génétique Médicale, INSERM U1211, Laboratoire MRGM, Université de Bordeaux, Bordeaux, France

***Correspondence**: Catherine Lejeune

[catherine.lejeune@u-bourgogne.fr](mailto:catherine.lejeune@u-bourgogne.fr)

**Table 1** Resources used (year 2018)

| **LABOR** | **Time**  **(minutes)** | **Wage (€/minutes)** | **Adjusted^d^ wage (€/minutes)** |
| --- | --- | --- | --- |
| **Step 1** *(Preparation of the pre-analytical step)* | | |  |
| Sending empty tubes (by post) and Excel files (by email) (1 engineer) | 30 | 0.97 | 1 |
| **Step 2** *(Pre-analytical step)* | | |  |
| Reception (1 laboratory technician) | 5.56 | 0.50 | 0.56 |
| Registration (1 secretary) | 8.06 | 0.48 | 0.54 |
| Validation (1 physician) | 1.96 | 1.03 | 1.16 |
| DNA extraction (1 laboratory technician) | 32.92 | 0.50 | 0.56 |
| Quality control (1 laboratory technician) | 29.63 | 0.50 | 0.56 |
| DNA aliquoting (1 laboratory technician) | 4.76 | 0.50 | 0.56 |
| DNA aliquots sending (by post) to the sequencing platform (1 biologist) | 4.94 | 1.03 | 1.16 |
| **Step 3** *(Analytical phase)* | | |  |
| **a. Sample preparation** | | |  |
| Sample reception/registration (1 engineer) | 19 | 0.97 | 1 |
| Content verification (1 laboratory technician) | 0.45 | 0.60 | 0.62 |
| Storage (1 laboratory technician) | 30 | 0.60 | 0.62 |
| Quantification (1 laboratory technician) | 14.48 | 0.60 | 0.62 |
| Quality control (1 laboratory technician) | 127.20 | 0.60 | 0.62 |
| Quality report sending to the laboratory team then selects samples for sequencing and notifies that plate is ready (1 engineer) | 45 | 0.97 | 1 |
| **b. Production** | | |  |
| Plate reception/registration (1 manager) | 15 | 0.97 | 1 |
| Library preparation (1 laboratory technician (2 for some steps)) | 419.75 | 0.60 | 0.62 |
| Sequencing (1 laboratory technician (3 for some steps)) | 623 | 0.60 | 0.62 |
| **c. Quality control and primary bioinformatics analysis** | | |  |
| Recovery of flowcell results and automatic production of fastq files (1 engineer bioinformatician) | 5 | 0.97 | 1 |
| First quality control and transfer to the computing center (1 engineer bioinformatician) | 15 | 0.97 | 1 |
| Quality control metrics (1 engineer bioinformatician) | 19.31 | 0.97 | 1 |
| Transmission of compressed files to the laboratory team (1 engineer bioinformatician) | 20 | 0.97 | 1 |
| **Step 4** *(Bioinformatics phase)* | | |  |
| Declaration of samples received (1 engineer bioinformatician) | 10 | 0.65 | 0.71 |
| Signature verification (1 engineer bioinformatician) | 2 | 0.65 | 0.71 |
| Data transfer to the computing center and storage (1 engineer bioinformatician) | 2 | 0.65 | 0.71 |
| Sequence and bioinformatics analyses (1 engineer bioinformatician) | 2 | 0.65 | 0.71 |
| **Step 5** (*Biological step*) | | | |
| Interpretation of variants and bibliographic research (1 biologist) | 12.76 | 1.03 | 1.16 |
| File preparation (1 hospital physician) | 14.87 | 1.03 | 1.16 |
| Multidisciplinary meeting: presentation and discussion (8 hospital physicians) | 2.77 | 1.03 | 1.16 |
| Report writing (1 hospital physician) | 17.85 | 1.03 | 1.16 |
| **DISPOSABLE MATERIALS AND REAGENTS** | **Quantities** | **Unit price (€ ATI)^a^** | **Unit price adjusted for the consumer price index (CPI) ^e^ (€ ATI)^a^** |
| **Step 1** *(Preparation of the pre-analytical step)* | | |  |
| Tube | 1 | 0.8 | 0.89 |
| Label | 1 |  |  |
| Envelope | 1 |  |  |
| **Step 2** *(Pre-analytical step)* | | |  |
| **DNA extraction** | | |  |
| Solution Lyse RBC | 15 ml | 0.18 | 0.2 |
| 1 Kit Reagents QiAamp | -^b^ | 8.87 | 9.90 |
| Rnase A | 20 µl | 0.11 | 0.12 |
| Square stickers | 4 | 0.04 | 0.04 |
| Round stickers | 3 | 0.03 | 0.03 |
| Pairs of gloves | 4 | 0.09 | 0.1 |
| Cones 20-200µl | 3 | 0.10 | 0.11 |
| Cones 0.5-20µl | 1 | 0.10 | 0.11 |
| Tubes 50 mL | 1 | 0.40 | 0.45 |
| Tubes 1.5mL for automat | 2 | 0.02 | 0.02 |
| Tubes GAD WR | 1 | 0.03 | 0.03 |
| Sample tube CB | 1 | 0.04 | 0.04 |
| **Quality control** | | |  |
| Agarose powder | 0.032 g | 0.28 | 0.31 |
| Buffer TBE = Tris-borate, EDTA | 3.34 ml | 0.02 | 0.02 |
| Buffer BET = Ethidium bromide | 0.03 µl | 0.00002 | 0.00002 |
| Charge buffer | 1 µl | 0.01 | 0.01 |
| Elution solution (Reagents Kits QiAamp Blood sample Minikit) | 0.12 µl | 0.002 | 0.002 |
| Molecular grade | 2 µl | 1.34 | 1.49 |
| Kit Qubit (Buffer + intercalant + standard) | -^b^ | 1.63 | 1.82 |
| Pairs of gloves | 8 | 0.09 | 0.1 |
| Strips | 1 | 0.74 | 0.83 |
| Tips starlab | 1 | 0.16 | 0.18 |
| Tips 0.5-20µl | 4 | 0.10 | 0.11 |
| Tips 2-20µl | 3 | 0.10 | 0.11 |
| Tips 20-200µl | 2 | 0.10 | 0.11 |
| Tubes qubit | 3 | 0.15 | 0.17 |
| Tubes 2mL | 1 | 0.04 | 0.04 |
| **Step 3** *(Analytical phase)* | | |  |
| **a. Sample preparation^c^** | | |  |
| Tips | - | 8.52 | 9.50 |
| Pair of gloves | - |  |  |
| 96 well plate for sample quantification | - |  |  |
| Optical film | - |  |  |
| Quantification kit | - |  |  |
| Fluorochrome | - |  |  |
| Sample plate | - |  |  |
| Plate for quality controls | - |  |  |
| Reagents for TapeStation 4200 | - |  |  |
| Diluent buffer | - |  |  |
| **b. Production^c^** | |  |  |
| Capture library preparation and QC | - | 166.31 | 185.48 |
| Sequencing kit and flow cell | - | 349.36 | 389.63 |
| **REUSABLE MATERIAL** | **Number** | **Unit price (€ ATI)^a^** | **Unit price adjusted for the consumer price index (CPI) ^e^ (€ ATI)^a^** |
| **Step 2** *(Pre-analytical step)* | | |  |
| **DNA extraction** | | |  |
| Pipette 2-20µl | 1 | 0.08 | 0.09 |
| Pipette 20-200µl | 1 | 0.08 | 0.09 |
| Distributor | 1 | 0.1 | 0.11 |
| Rack for 1.5 mL tubes | 1 | 0.01 | 0.01 |
| Rack for 50 mL tubes | 1 | 0.004 | 0.005 |
| **Quality control** | |  |  |
| Rack | 2 | 0.01 | 0.01 |
| Bins for intercalant ADN | 2 | 0.005 | 0.01 |
| Variable gap pipette 5-50µl | 1 | 0.08 | 0.09 |
| Pipette 500µl-5mL | 1 | 0.08 | 0.09 |
| Multichannel pipette 0,5-10µl | 1 | 0.34 | 0.38 |
| Pipette 0.5-10µl | 1 | 0.08 | 0.09 |
| **Step 3** *(Analytical phase)* | | |  |
| **a. Sample bank preparation** | | |  |
| Pipette | 1 | 0.003 | 0.004 |
| Multichannel pipette | 1 | 0.01 | 0.01 |
| **b. Production** | |  |  |
| Single channel pipette | 6 | 0.01 | 0.01 |
| Multichannel (8) pipette | 2 | 0.04 | 0.04 |
| Multichannel (12) pipette | 1 | 0.08 | 0.08 |
| **EQUIPMENT** | **Number** | **Unit price (€ ATI)^a^** | **Unit price adjusted for the consumer price index (CPI) ^e^ (€ ATI)^a^** |
| **Step 2** *(Pre-analytical step)* | | |  |
| **DNA extraction** | | |  |
| Centrifuge for 50 mL tubes | 1 | 1.26 | 1.40 |
| Centrifuge for 1.5 mL tubes | 1 | 1.26 | 1.40 |
| Vortex | 1 | 0.09 | 0.1 |
| Qiacube | 1 | 2.96 | 3.3 |
| **Quality control** | | |  |
| Qubit | 1 | 0.34 | 0.38 |
| Multiscan | 1 | 0.74 | 0.83 |
| Geldoc | 1 | 0.74 | 0.83 |
| Electrophoresis tank | 1 | 0.21 | 0.23 |
| Generator | 1 | 0.07 | 0.07 |
| Balance | 1 | 0.04 | 0.05 |
| Magnetic agitator | 1 | 0.02 | 0.02 |
| Centrifuge | 1 | 0.59 | 0.66 |
| **Step 3** *(Analytical phase)* | | |  |
| **a. Sample preparation** | | |  |
| Centrifuge | 1 | 0.04 | 0.04 |
| Magnetic agitator | 1 | 0.004 | 0.004 |
| Fluorescence plate reader | 1 | 0.16 | 0.18 |
| Pipetting robot | 1 | 0.46 | 0.52 |
| PCR Device | 2 | 0.02 | 0.03 |
| TapeStation 4200 | 1 | 0.16 | 0.18 |
| Electrophoresis tank | 2 | 0.003 | 0.003 |
| InGenius LHR | 1 | 0.03 | 0.04 |
| **b. Production** | | |  |
| Covaris | 1 | 3.77 | 4.21 |
| Bravo | 2 | 3.16 | 3.52 |
| LabGX | 1 | 0.88 | 0.98 |
| Thermocycler | 1 | 0.13 | 0.14 |
| Qbit | 1 | 0.06 | 0.06 |
| Speed Vac | 1 | 0.34 | 0.38 |
| Janus | 1 | 1.72 | 1.92 |
| CBOT | 2 | 0.85 | 0.95 |
| Hiseq4000 | 1 | 29.61 | 33.02 |
| **c. Quality control and primary bioinformatics analysis** | | |  |
| Computer | 1 | 0.17 | 0.19 |
| Storage | 60 go | 0.33 | 0.37 |
| Computing hours | 300 | 0.03 | 0.04 |
| **Step 4** *(Bioinformatics phase)* | | |  |
| Computer | 1 | 0.03 | 0.03 |
| Storage | 20 go | 0.15 | 0.16 |
| Computing hours | 50 | 0.004 | 0.004 |
| **MAINTENANCE** | | |  |
| **Step 2** *(Pre-analytical step)* | **Number** | **Unit price (€ ATI)^a^** | **Unit price adjusted for the consumer price index (CPI) ^e^ (€ ATI)^a^** |
| **DNA extraction** | | |  |
| Qiacube | 1 | 7.32 | 8.16 |
| **Step 3** *(Analytical phase)* | **Number** | **Unit price (€ ATI)^a^** | **Unit price adjusted for the consumer price index (CPI) ^e^ (€ ATI)^a^** |
| **a. Sample preparation** | | |  |
| Centrifuge | 1 | 0.004 | 0.004 |
| Fluorescence plate reader | 1 | 0.25 | 0.28 |
| Pipetting robot | 1 | 0.25 | 0.27 |
| PCR device | 2 | 0.01 | 0.02 |
| TapeStation4200 | 1 | 0.13 | 0.15 |
| **b. Production** | | |  |
| Covaris | 1 | 2.35 | 2.62 |
| Bravo | 2 | 2.03 | 2.26 |
| LabGX | 1 | 0.45 | 0.50 |
| Thermocycler | 1 | 0.07 | 0.08 |
| Janus | 1 | 1.13 | 1.26 |
| CBOT | 2 | 0.42 | 0.47 |
| Hiseq 4000 | 1 | 12.90 | 14.38 |
| **Step 4** *(Bioinformatics phase)* | **Time**  **(minutes)** | **Wage (€/minutes)** | **Adjusted wage (€/minutes)^d^** |
| Pipeline maintenance (1 engineer) | 25.8 | 0.65 | 0.71 |

^a^ ATI = All Taxes Included

^b^ Global price was provided by the genetic team

^c^ Global price was provided by the sequencing platform

^d^ Wages were increased from salary enhancement measures taken from 2020 in France (Segur law and a 3.5% increase according to the status)

^e^ Prices were increased by 11% (rate calculated from January 2018 until October 2022 *via* the Consumer Price Index -CPI- issued from the Insee)
